# Flip flops, dress clothes, and no coat: clothing barriers to children's physical activity in child-care centers identified from a qualitative study

**DOI:** 10.1186/1479-5868-6-74

**Published:** 2009-11-06

**Authors:** Kristen A Copeland, Susan N Sherman, Cassandra A Kendeigh, Brian E Saelens, Heidi J Kalkwarf

**Affiliations:** 1 Division of General and Community Pediatrics, Cincinnati Children's Hospital Medical Center, Cincinnati, OH, USA; 2 Department of Pediatrics, University of Cincinnati College of Medicine, Cincinnati, OH, USA; 3 SNS Research, Cincinnati, OH, USA; 4 Seattle Children's Hospital Research Institute & Department of Pediatrics, University of Washington, Seattle, WA, USA

## Abstract

**Background:**

Three-quarters of 3-6 year-old children in the U.S. spend time in childcare; many spend most of their waking hours in these settings. Daily physical activity offers numerous health benefits, but activity levels vary widely across centers. This study was undertaken to explore reasons why physical activity levels may vary. The purpose of this paper is to summarize an unexpected finding that child-care providers cited was a key barrier to children's physical activity.

**Methods:**

Nine focus groups with 49 child-care providers (55% black) from 34 centers (including inner-city, suburban, Head Start and Montessori) were conducted in Cincinnati, OH. Three independent raters analyzed verbatim transcripts for themes. Several techniques were used to increase credibility of findings, including interviews with 13 caregivers.

**Results:**

Two major themes about clothing were: 1) children's clothing was a barrier to children's physical activity in child-care, and 2) clothing choices were a significant source of conflict between parents and child-care providers. Inappropriate clothing items included: no coat/hat/gloves in the wintertime, flip flops or sandals, dress/expensive clothes, jewelry, and clothes that were either too loose or too tight. Child-care providers explained that unless there were enough extra coats at the center, a single child without a coat could prevent the entire class from going outside. Caregivers suggested several reasons why parents may dress their child inappropriately, including forgetfulness, a rushed morning routine, limited income to buy clothes, a child's preference for a favorite item, and parents not understanding the importance of outdoor play. Several child-care providers favored specific policies prohibiting inappropriate clothing, as many reported limited success with verbal or written reminders to bring appropriate clothing.

**Conclusion:**

Inappropriate clothing may be an important barrier to children's physical activity in child-care settings, particularly if the clothing of a few children preclude physical activity for the remaining children. Center directors and policy makers should consider devising clear and specific policies for the types of clothing that will be permitted in these settings so that children's active play opportunities are not curtailed. To enhance compliance, parents may need education about the importance and benefits of active play for children's development.

## Background

Three-fourths of U.S. children aged 3 to 6 years are in some form of non-parental child care; 57% are enrolled in child-care centers, nursery schools, or preschools [1]. Many children spend most of their waking hours in child care [2]. Daily physical activity is essential for healthy childhood growth and development [3-6], and confers numerous health, cognitive, and mood benefits to young children [7-9]. And yet, many children in child care are not meeting daily recommendations[10] for physical activity as recent studies found that children spend most of their time in child care being sedentary [11,12]. Furthermore, the amount of physical activity that children obtain in child-care varies widely across different centers [11-13].

Given the large number of children in child-care and the numerous benefits to physical activity, policy-makers and researchers in physical activity and public health have turned their attention towards child-care centers as a potential venue for health promotion and childhood obesity prevention [14,15] These reports called for more detail about the problems and potential solutions in order to inform strategies to increase children's physical activity in child care.

Recent quantitative studies have investigated reasons why physical activity levels may vary among centers, including differences in the physical environment including fixed and portable play equipment (e.g., balls) [16,17], the center's policy related to active and inactive time, and supporting physical activity [17,18] the educational quality of the child-care center [16,18], and teacher training and education [17,18]. Since time spent outdoors has been found to be associated with activity levels [19-21], several investigators have sought to determine if increasing the amount of time scheduled for outdoor play could predict higher physical activity levels [16-18,22]. In two of four studies, [16,22] increased time spent outdoors was not associated with increased levels of physical activity. None of these quantitative studies considered that a child's clothing might restrict physical activity in spite of ample outdoor play equipment and time scheduled for outdoor play, or that a center's policies dictating what types of clothing may be worn might have an effect on children's physical activity; as these potential issues have not been widely reported in the physical activity literature.

Experts in child-care physical activity have categorized the field as still being in its infancy, and have called for innovative and out-of-the box approaches [23]. In new fields of inquiry, qualitative research can be helpful in: identification of salient themes and constructs, understanding the meaning and context of new constructs, identification of unanticipated phenomena or influences, and development of causal explanations of quantitatively observed phenomena [24].

The larger study, of which this paper is a part, was designed to employ these benefits of qualitative inquiry to explore reasons why physical activity levels may vary across different child-care centers. The purpose of this paper is to describe two major themes from our findings that that were unexpected based on our review of the relevant literature: 1) that child care providers cited children's clothing as an important barrier to children's physical activity, and 2) children's clothing contributes to considerable conflict between parents and child-care providers.

## Methods

### Study overview

Qualitative research methods were chosen to explore child-care providers' perceptions of facilitators and barriers to children's physical activity in child-care centers. Focus groups were chosen as this method promotes spontaneous discussions and interaction among group members, and is particularly effective in eliciting normative beliefs [25-27]. We used one-on-one interviews to confirm the credibility of our focus group findings ("member-checks"[24]), and to explore more sensitive topics that focus group participants may not have felt comfortable discussing in a group setting[25]. We conducted nine focus groups with child-care providers between August 2006 and June 2007 and 13 one-on-one interviews in the spring of 2008; nine interviews were member-checks with former focus group participants, 4 interviews were with individuals who were selected in the original sample for the focus groups but were unable to attend any of the sessions. These were approved by the institutional review board (IRB) at Cincinnati Children's Hospital Medical Center,

### Sampling and recruitment for focus groups

Participants were eligible to participate if they had worked, in the past three years, as a child-care provider or teacher for a full-day child-care center or preschool in Hamilton County, Ohio (Cincinnati area). No more than one participant per child-care center was eligible to attend each focus group, so that there was heterogeneity of experiences. This also minimized the chances that certain focus group members knew one another, which could make other focus group members feel uncomfortable and hamper the free-flow of ideas[27]

Maximum variation sampling[25,28] was used to select purposively a heterogeneous sample of child care teachers, thereby securing a small sample of great diversity. The strength of this strategy is that identification and description of emerging themes takes on greater importance as it cuts across greater variety of participants. A limitation of this method is that it potentially takes longer to reach saturation of themes. Specifically, we targeted recruitment of teachers from different ethnic backgrounds and with a range of years of experience. Moreover, we recruited those who worked in both suburban and urban centers, those which served both low-income and upper-income children, and incorporated a range of philosophies and affiliations (e.g., Montessori, Head Start, church-affiliated, YMCA, worksite- or University-affiliated, and corporate/for-profit centers). To accomplish this, we enlisted the help of several local agencies devoted to child-care continuing education, quality, and safety: the local child care resource and referral agency 4C, the Cincinnati Association for the Education of Young Children, Cincinnati Community Action Agency (Head Start), the United Way, three local universities with early education programs, and the American Academy of Pediatrics Healthy Child Care Ohio program. Respective agencies posted announcements on their websites, in their offices, in their written and electronic newsletters, and also distributed personal invitations to participate. A minimum expected sample size was specified at the outset of data collection, and we continued to sample to the point of "theoretical saturation,"[25,27] when no new information was forthcoming from participants in additional focus groups. Determination of thematic saturation was reached by consensus, and recruitment for additional focus groups was suspended.

Seventy-two child-care providers responded to the various recruitment methods (the majority had seen a flier or had heard about the study through the local resource and referral agency). Four were ineligible to participate (because they provided care in their own homes), three were unable to be scheduled in any focus group or interview, three later indicated they were not interested, three were lost to follow-up (no working phone number), six cancelled, and four were no-shows; leaving 49 participants in nine focus groups.

### Data collection

All focus groups were held in a private conference room in Cincinnati Children's Hospital Medical Center in the early evening. The focus groups averaged 1.5 hours in duration, and were audio-taped and transcribed verbatim. All focus groups were moderated by one of the investigators (S.N.S), an experienced focus group facilitator. The principal investigator (K.A.C) attended all focus groups to take notes and record non-verbal cues (body language, etc.). All participants provided verbal informed consent to participate and completed an anonymous brief demographic questionnaire. Light refreshments were served and all participants received $25 reimbursement for their time.

The focus group topic guide used broad, open-ended questions designed to elicit child-care providers' perceptions of facilitators and barriers to children's physical activity in child-care settings. Examples of the questions and probes used that elicited the themes presented in this manuscript are in the Appendix. Broad questions were followed up by more specific probe questions as necessary. The attendance of two study investigators at each session allowed for preliminary data analysis concurrent to data collection [29], which was useful both for knowing when to suspend recruitment for focus groups and for making necessary adjustments to the topic guide. The topic guide was modified slightly over time in an iterative process to accommodate new issues raised by participants, and to clarify issues brought up in earlier groups. After the ninth focus group, two investigators (KAC and SNS) reached a consensus that no new information or themes were emerging from discussions ("theoretical saturation" [25,27]), so recruitment for focus groups was terminated as originally planned.

One-on-one interviews were conducted in April 2008 with 9 focus group participants and 4 new participants who had been selected as part of the initial sample but were unable to attend any of the sessions. The audio-taped sessions took place in either the interviewee's home or private office at a child-care center and lasted approximately 1 hour. The interviewer reviewed with the interviewee an expanded version of the code framework that had been derived from analysis of focus group transcripts (see next section on data analysis) and outlined the major themes identified by the research team. Interviewees were given an opportunity to expand on or differ with this listing of themes. All interviewees provided written informed consent to participate and received $25 for their time.

### Data analysis

We used an inductive approach [28] whereby we looked for patterns, themes and categories in our data, without applying any pre-conceived constructs, hypotheses, or theories to the process of interpretation. Thus we identified, categorized, coded and labeled the primary patterns of ideas that emerged from the verbatim comments contained in the transcripts of our focus groups. Using an organizing style of analysis, 3 investigators (KAC, SNS, CAK) each independently read transcripts thoroughly, and generated a set of initial codes or categories of ideas. Next, the group used focused coding to identify, describe and prioritize the repeating ideas and patterns of ideas as major themes. A coding framework (or codebook) was developed which contained individual codes for each of the themes identified by the team. In the next step each of the coders used the codebook to independently code a representative transcript. The group met once again to resolve problems that arose in applying the codebook and to make revisions as needed. The three investigators continued to independently code the remaining transcripts, meeting after each one to resolve any differences in coding by consensus. Any discrepancies were resolved by another reader. NVivo 7 software (QSR International) was used to manage the qualitative data.

None of the interview participants who had participated in focus groups (member checks), nor the four interview participants who had not previously participated, disagreed with the team's identification and analysis of the major themes of the study (including the two main themes presented in this paper). Furthermore, no new major themes emerged from the one-on-one interviews. Interview subjects provided additional insights and supporting experiences, which were used to assist in the interpretation and analysis of our data.

### Interviews and Data Credibility

Our research design which included maximum variation sampling, multiple data collection methods and our diverse team of investigators enhanced the credibility of the data analysis [28,30]. Our sampling strategy enabled us to identify and recruit participants with a wide variety of experiences who represented a cross-section of types and locations (suburban vs. urban) of child care centers. Our team was composed of multiple investigators with different types of academic training (pediatrics, nutritional epidemiology, social science research, and psychology), and one (CAK) had experience working in a child-care center. By using a collaborative approach, multiple perspectives were voiced and discussed throughout the analysis phase. Coding occurred independently, and all final codes were agreed upon by group consensus. Member checking was carried out through one-on-one interviews with participants and non-participants of prior focus groups. Member checking provided an opportunity to obtain new information that might not have been forthcoming in a group setting and it enabled us to verify the information already collected as well as clarify and crystallize our interpretation and data analysis. Finally, further triangulation of our data collection and analysis occurred in an unplanned method: preliminary results of this study were reported at a scientific meeting in May 2008 and covered electronically in a wellness blog in *The New York Times*(Tara Parker-Pope, May 6, 2008) [31]. All 132 blog postings posted within 1 week were reviewed by the authors, but revealed no new themes or comments that had not already been mentioned by focus group and interview participants.

## Results

### Sample demographics

The demographic characteristics of the 49 focus groups participants and 13 interviewees were reflective of an inner-city American child-care work force [32] (Table 1). All but one of the participants were female; most had at least some college education. Roughly half of participants identified themselves as black/African-American. Level of experience in the field varied from less than one year to 37 years. While most participants currently worked with preschool-aged children, 26% reported primarily working with infants and toddlers under the age of 3, and the remainder primarily worked with school-aged children (8%), floated between different age-groups (6%), or currently served in a supervisory role (6%). Three focus group participants and four interviewees were center directors.

**Table 1:** Sample Demographics^a^

	Focus Groups N = 49	Interviews^b^ N = 13	TOTAL^c^ N = 53
**Gender**			
Female	48 (98)	13 (100)	52 (98)
Male	1 (2)	-	1 (2)
			
**Race**			
Black	27 (55)	7 (54)	28 (53)
White	22 (45)	6 (46)	25 (47)
			
**Age in years**, *mean (SD)*	39 (11)	44 (10)	40 (11)
			
**Education**			
Did not graduate high school	2 (4)	-	2 (4)
High school diploma/GED	3 (6)	1 (8)	3 (6)
Some college/assoc. degree	27 (55)	6 (46)	29 (55)
Bachelor's degree	10 (20)	4 (31)	11 (21)
Graduate school	7 (14)	2 (15)	8 (15)
			
**Have children of their own**	37 (75)	12 (92)	41 (77)
Used child care for own children	28 (76)	9 (75)	31 (76)
			
**Years worked as child care provider**, *mean (SD)*	13 (9)	12 (11)	13 (9)
			
**Hours worked per week**			
0-30	10 (20)	5 (38)	12 (23)
31-40	27 (55)	7 (54)	29 (55)
>40	12 (24)	1 (8)	12 (23)
			
**Age group cared for**			
Infants/toddlers	13 (27)	2 (15)	14 (26)
Preschool	26 (53)	5 (38)	26 (49)
Kindergarten/school-age	4 (8)	1 (8)	4 (8)
Floater (age group varies)	3 (6)	1 (8)	3 (6)
Supervisor	3 (6)	4 (31)	6 (11)

^a ^All data is presented in format of n (%) unless otherwise indicated.

^b ^Interview participants consisted of 9 child-care providers who had participated in interviews ("member checks") and 4 child-care providers who had been recruited, but unable to participate in focus groups.

^c ^Total sample includes 49 participants of focus groups and 4 child-care providers who only participated in interviews.

Focus group participants came from 34 centers that were well-distributed geographically throughout the county including 12 centers located outside the city limits and 13 centers located in low-income U.S. Census tracts (median income is less than 50% of median income for Cincinnati's metropolitan statistical area (CMSA)). The types of child-care centers were also diverse, including five Montessori, six Head Start, two church-affiliated, two YMCA, four worksite- or University-affiliated, and three corporate/for-profit centers. The four interview participants who had not been able to participate in the focus groups came from three additional centers.

### Themes related to inappropriate clothing

When asked to describe what barriers existed in child-care centers to prevent children's physical activity, virtually all participants cited barriers related to children's clothing (Table 2). Other barriers were also mentioned and will be presented in a separate paper. Inappropriate clothing and conflict resulting from this clothing were determined to be major themes for two reasons. First, discussions about inappropriate clothing and conflict related to clothing resulted spontaneously from general questions about barriers (e.g., "What keeps children from being active?") and non-specific probes (e.g. "Tell me more about that"). Second, these discussions about clothing issues were mentioned by virtually all participants. These major themes will be presented in four sections: 1) a listing of categories of inappropriate clothing items, 2) suggested reasons for inappropriate clothing, 3) teacher-parent conflict related to clothing, and lastly 4) possible solutions to conflict and clothing barriers. Inappropriate clothing items are presented in order of most commonly to least commonly mentioned during the focus groups: weather-related, flip-flops and other footwear, dress clothes, jewelry, and ill-fitting clothes.

**Table 2:** Clothing Barriers and Possible Solutions

Barrier	Problem/Example	Possible solutions
No coat (or hats, gloves, or boots) in winter	1-2 children without coats can prevent the entire class from going outside	Parent reminders Center keeps extra coats, gloves, etc. for loan Parents leave extra clothes in child's cubby Bringing the snow inside Use the muscle room

Over-dressed for temperature	Center policies require children wear all clothing that their parents send.	Change policies to be more flexible Parent education/clarify parent requests

Flip flops	Do not protect feet from mulch/rocks Do not support feet well when climbing Fall off easily, shoes can get lost	Parent education Keep extra pair of gym shoes at school Policies restricting flip flops

Nice clothes	Kids can get dirty Clothes can be ruined	Parent education Developing a rapport with parents:

Jewelry	Small parts are choke-ables for toddlers Large pieces can get caught in equipment Can get lost	Teachers remove item, parent education

Improperly fitting clothes: too lose/too tight	Loose fitting clothing can get caught in equipment, or fall off Tight clothes (e.g., skirts) can restrict ability to run	Parent education

### Categories of inappropriate clothing

#### Weather-related

Clothes ill-suited for the weather comprised the most frequently encountered clothing issue. In the winter, teachers reported often having children arrive without coats, hats, gloves and/or boots.

¶1 "If they don't have a hat and gloves, then it would impede them from going outside."

¶2 "You have the times where it's cold and they might come in sandals or all the other kids have on hats and gloves and big coats, and they have on a windbreaker."

In the wintertime a single child's inappropriate clothing could cause problems for the entire class. Participants noted they could leave the child inside with another teacher or director while taking the rest of the class outside but only if they had adequate staff. Otherwise only a few children dressed improperly can prevent the entire class from going outside.

¶3 "Since I don't have anybody to watch the children who aren't dressed properly, I can't take the class out and leave one child" [that's dressed improperly]

¶4 "If you have some that can't go out, then none can go out."

But moderate temperatures also presented problems at some centers as participants stated many centers have policies that the child must wear every layer of clothing the parent sent with them. Others stated that they would interpret a parent bringing a coat as implicit orders for their child to wear the coat. Thus children could be over-dressed for the temperature, which could impede their ability to participate in physical activities.

¶5 "I have one child ... He came to school with an undershirt a thermal shirt and a sweat shirt all winter and a coat zipped up and maybe some type of sweater. You know, I think she had him over dressed. And he would come in [he says] 'I I I I take off my shirt?' I can't let you take off your shirt. 'I so hot.' I say I understand."

¶6 "We have Parents that, it's 65 degrees outside and they have hats and mittens. And we're like 'Why?' They still have their winter coats on. And we're like,. 'I hate putting this on you'."

¶7 "'You have to.' I always say, 'What your mom sent you in, that's what you are going to wear.' I sit there and sweat with them."

#### Flip flops & inappropriate footwear

The next most commonly mentioned problem clothing item was inappropriate footwear, including flip flops and sandals and shoes with slippery soles. Teachers explained that these shoes did not provide adequate support for running and climbing activities. Additionally, teachers found that flip flops could come off easily when running or walking briskly.

¶8 "We like them to wear gym shoes because of the activity level, you know, playing and stuff. Even in the summer, we ask them, 'Don't wear sandals and flip flops and stuff cuz they come off their feet and they're just not very safe'."

9¶"When they're running, the flip flop either comes off or that foot ends up going over the top and scraping the ground."

¶10 "Field trips, you know ... They don't say nothing for a while and you look back and they're crying, (we ask) 'Why are you crying?' [The child says] 'My shoe (is) way back there'."

Participants also explained that flip flops did not provide adequate protection against common playground surfaces such as mulch and gravel.

¶11 "They wear them in the rocks and every 2 seconds you're taking the shoes off... It hurts. It really is detrimental to the children because they can't play like they should be playing."

¶12 " [Parents will] bring them in flip-flops and we have a mulch playground too and they're--the children are constantly, 'I have mulch in my shoes.' So, they're always taking their shoes off and dumping the mulch out. Five minutes later it is back in their shoes."

Despite the commonly-understood problems that flip flops presented to children's physical activity, only a few teachers reported center policies restricting the use of flip flops, and usually these policies only applied to days with field trips. Many wished their centers prohibited flip flops at all times.

#### Dress clothes

Another type of clothing that participants saw as a barrier to children's physical activity was a "nice" or special outfit in which the parent instructed the child or the teacher not to get dirty.

¶13 "I've had problems with parents telling the kids, 'Don't get dirty!' because of what they have on. So that's I guess a pet peeve of mine... They're going outside. They are going to play. They are going to be on the floor. They are going to be, you know, *children*."

¶14 "One of the parents ... she sent her child first time wearing shoes and from the scooter, she had two holes in her brand new shoes. I tried to explain to her that we can't pay for them. What do you want us to tell her, 'You can't get on the bike?' She said, 'Yes, let her cry."'

¶15 "We sometimes have children who when they go outside they will sit on the bench and when you ask them why they're sitting there, they say, 'My mommy said I couldn't get dirty."'

#### Jewelry

Several participants characterized jewelry as a potential barrier to children's physical activity, because it could get caught in equipment, or if lost it could pose a choking hazard to younger children. Many participants reported having policies that expressly prohibited wearing jewelry, yet several found children would still arrive with prohibited items. A child arriving at school wearing jewelry and the implicit parental request to teachers to keep track of this jewelry was a significant source of conflict between parents and child-care providers.

¶16 "I even have kids come with chains, necklaces, bracelets. I don't like that. The little gum ball rings. Because with the toddlers in the center, if you drop that ring in the muscle room and then the toddlers come in after us, and they're putting that in their mouth....There is no reason for him to have on a gold watch. It's no place for it."

¶17 "Bangles, they got bracelets all the way up to here, getting caught in hair...... One little kid was wearing this long gold chain and the other kids were choking him by the neck--bling, bling--I was like, 'Daddy, could you please, I know you like your son to look nice but this is for the weekend not for school.' Most of them be pretty nice about it but sometimes they still come with the earrings and bracelets and stuff."

¶18 " [We say] 'Please, do not send your child here with $300 chain on his neck he's 3, 4, 5!' You know, I said I am not gonna be responsible for keeping up their jewelry. Most of them [parents] listen and say 'OK, we won't,' but, why would you send your child to preschool with a $500 chain on?! I don't understand that!"

#### Ill-fitting clothes

Lastly, participants mentioned that ill-fitting clothing could impair physical activity. For instance, long flowing skirts could get caught in climbing equipment; loose-fitting pants and shoes could hamper a child's ability to run. Excessively tight-fitting clothing could also restrict a child's activity.

¶19 "A skirt and long dresses they wear when they're climbing, they're taking a step and their foot gets caught. It's really dangerous."

¶20 "We really deal with the clothing and stuff. You are starting to see more and more children, like wearing the big pants. They can't run and play, not only outside but inside either. I think it's really stopping them from doing things."

¶21 "We have had children come to school with pants hanging down, too big. Boys' pants hanging down, too big, no belts. Shirts and stuff that look like big brother's shirt or something too big, they're way too big. They are too big to maneuver on the climber."

¶22 "The very tight skirts and very, very short skirts. Those poor little girls sit there and they don't want to play, you know."

### Reasons for inappropriate clothing

Teachers speculated several reasons why children in this age group might come to school inappropriately dressed. Explanations included: a rushed morning routine, a "car culture," the child's clothing preferences, practical difficulties finding clothes that fit in rapidly growing preschoolers, first-time parents concerned with their child's appearance, insufficient income to afford proper clothes, or deliberate parent clothing choices.

Teachers acknowledged that parents may simply forget to bring appropriate winter clothes in the rush to get out the door.

¶23 "Sometimes parents are in a rush in the morning and in the cold weather they don't send mittens or a hat. You know, and that's discouraging."

¶24 *Participant*: "At my center the parents forget the jackets."

*Another participant in response*: "We have had that. They forget the gloves."

¶25 "It's just up to the teachers kind of to send notes home and just ask them not to wear them [flip flops]. But, the kids get it in their head that that's what they want to wear too and I'm sure the parents are dealing with whatever they need to deal with in the morning to get them going."

A few hypothesized that the American "car-culture," in which children spend most of their day indoors or being transported from place to place inside a vehicle, may contribute to this.

¶26 "There is another thing about children in the 'car culture' that sometimes they are not dressed appropriately for outside. They go from their heated house to their heated garage in their heated car (*laughter*) and get carried into school, sometimes they even...it's like, [the parents say upon arrival] "Oh! I didn't know he didn't even have his shoes on!!"

¶27 "Sometimes they don't have one [a coat] and sometimes they are in a car so they don't think that you are supposed to have a jacket. They're just coming in the building and when they pick them up, they're just going back in the car. So that's how they'll do."

¶28 "A lot of parents feel it's too cold outside anyway [to go outdoors to play]. They're just getting out the car and going in the building. They don't have to be too clothed and then there goes the argument."

In some cases, participants speculated the flip-flops or the ill-fitting clothing item could be what is in fashion or one of the child's favorites (e.g., a big-brother's shirt), or an item they can put on themselves.

¶29 "Maybe it's the child who dressed themselves that day and they want to wear their fancy shoes."

¶30 "I had one little boy who wore his cousin's shoes, like loafers, but they were like three sizes too big and his mom said, 'Well, you know he loves them, da da da...' I said, 'Well, you know he can't walk in them. And he can't go out without shoes. I am just telling you, he's in the muscle room and he's falling down.' So make a choice. Either tell your child that he has shoes for school and he can't wear those, you know, or something."

A few participants also noted some developmental or age-specific reasons why preschoolers' clothes may be over-sized. It may be difficult to find clothes that fit properly for very thin preschool-aged children with "no hips." They added that belts may be impractical for children at this age who are still mastering self-toileting skills.

¶31 "I totally hear you with the belts--But if there is no belt and they're running and their pants are heading south! (*laughter, agreement*). That's so frustrating for these poor little girls and boys who have absolutely no hips. It's just, their pants are just falling down. In the preschool age, you want exactly what you said, you want easy access for the bathroom. Cuz if they're newly potty trained, they don't have any time, but...if they are dressed appropriately for the potty training, sometimes that's inappropriate for the outside."

As for children who come to school in dress clothes instead of play clothes, one teacher found this to be more of an issue among first-time parents, who were more likely to be concerned with preserving a new outfit.

¶32 "Usually it's first time, anxious parents who think their kids need to look a certain way all the time."

Several participants added that lower-income parents may not own enough clothes to leave extra clothes at the center. These parents may not be able to afford coats--much less snow pants or boots that may only be used a few days out of the year.

¶33 "We ask them to have extra clothes but there are many families that are just struggling to put the clothes on the children's back in the first place that can't have the extra clothes. So if they were to go out when it was raining, we wouldn't have anything to change them into for the rest of the day."

¶34 "The dressing the children, because we're from a low income [neighborhood], we work very hard to find every child a coat, hat and gloves. If they don't have a hat and gloves, then it would impede them from going outside. But when I was little, we used to have snow pants so even when it snowed, you could go outside. Children just don't seem to have snow pants anymore so if there is snow outside, then they can't go cuz no matter what the temperature, cuz they would be getting wet."

While teachers acknowledged several irreproachable and pragmatic reasons why children may arrive at the center in inappropriate clothing, some suspected that parents' ostensible forgetfulness may indicate an underlying failure to recognize the value of physical activity and outdoor time.

¶35 "The parents who don't see physical play as appropriate send them in inappropriate shoes, and flip flops, the stiletto 4 year old sandals."

¶36 "The parents don't really say anything until their child comes home. He didn't have that extra pair of clothes for whatever reason and they're all muddy. I am a teacher that likes to get down in the mud and work with some of the kids. I have had those parents who were kind of like, 'Oh, you're all muddy!' (*said with sarcastic and fake enthusiasm)*. And it stinks because it's a good learning experience. There is so much you can talk about: living creatures, worms. It's hard when the kids can't get muddy because they don't have an extra pair of clothes or the parents don't want to deal with washing clothes."

Some participants had witnessed and others suspected that parents intentionally took their child's coat with them to prevent outdoor play.

¶37 "I had an experience with parents when they bring the child in with a coat but take the coat with them when they go to work!"

Moderator: Do you ask them why they do that?

" [The parents say] 'Oh it was just in my hand and I forgot I had it.' [I say] 'Well, why didn't you bring it back when you realized you still had it in your hand?' ... They come up with some excuses."

¶38 "Our parents will take their child's coat with them if they don't want their child to go outside! I had two parents that took their child's coat because they didn't want them to be outside. I had a surprise for them because I keep extra coats! (*laughter*) So they will be outside."

### Teacher-parent conflict

In discussions about inappropriate clothing and jewelry, there was a recurring theme of tension between parents and teachers. Inappropriate clothing items seemed to act as a lightning rod for drawing out sparks of conflict between parent and teachers.

¶39 "It's like, How did you not notice? It's winter out here! There's snow, it's cold! How did you not notice he didn't have on his coat? Oh, he left it in the car or "oh, he left it at the house. How? You have your coat on. Why can't he have his coat on?! So it's like, you know, some parents are kind of like... I don't know!"

¶40 "We had a problem with jewelry. We have little girls and they would bring in necklaces. And I'm like, 'We are gonna go on the climber so why would you put [that on your child]? We are gonna get on the bike, why would you put?' I will be working in the toddler room most of the time so therefore there are a lot of little toddlers and they just want to touch. When her necklace gets broke, they're like 'Well I paid this much' And I'm like 'Well, then you should not have put it on her to come here.' That's all I can say about it! We had an incident where a little boy took off his earring and dropped it down in the heater! (*laughter*) So the mother is mad at the teacher and she wants to fight the teacher because she thinks the teacher dropped the earring. (*laughter*) Why would she drop the earring down in there?!"

Participants occasionally questioned parents' wardrobe decisions and resented parents' requests to be responsible for changing the child into play clothes so that the nice clothes could be preserved unharmed.

¶41 "We had a little girl two weeks ago in *corduroy*. It's 93 degrees outside. Corduroy and a tank top! What are you thinking? And Sandals? Anytime you can wear sandals, that means you don't need corduroy! (*laughter*) One might think."

¶42 "I've had a parent--and it was crazy--but I had a parent who brought play shoes! For when we went outside--we're gonna have to switch shoes!! (*much laughter*). You know, he came in the good shoes and she brought the play shoes so the good shoes wouldn't get messed up. So that was like, Really? You might as well just send him in the play shoes! (*agreement*) It's just weird, I guess it's just like parent's preferences. When it comes to people's kids, it's like you can't really tell them too much. It's like 'This is my child'!"

Seemingly unreasonable parent requests led another teacher to describe why she appreciates state licensing guidelines for child care:

¶43 "Mostly, it [the guidelines] allows us to be able to let the parents know we have limits. Bottom line. Because they ask us to do the strangest things. Yeah, That's the biggest thing for me, letting them know that we have limits to what we are allowed to do, just like you do on your job. They look at us like we are babysitters. To them, a lot of them. But I have to tell them, No, I'm an educator."

The discussions about children's clothing brought out some of the most heated sentiments among participants about parents. The following comments were elicited by a discussion about children arriving in flip flops, without coats, and a parent's request to preserve a new outfit.

¶44 "That's the biggest issue, for real. The parents are the biggest issue! They are worse than the kids!"

¶45 "You can tell which parents are gonna be like that because they come to the door. They put that vibe out to you. And you're like, OK, that's going to be a tough one! You can pick up on them."

### Possible solutions to clothing barriers and building a rapport

In light of the amount of conflict that clothing issues could incite between teachers and parents, participants offered several pragmatic solutions that involved minimal to no contact with parents. (Table 2). Many teachers kept extra coats and/or gloves at the center that children could borrow as needed. In centers with muscle rooms or gross motor rooms (about half of focus group participants reported having these), teachers could do physical activities indoors on days that children were dressed inappropriately for the weather.

¶46 "If it snows, sometimes I will take a bucket outside and get a bunch of snow and put the snow on the water table. Just like the sand, they will bury things in the snow. You can kind of bring the outside in."

Yet most solutions required some form of interaction with the parents, since participants acknowledged that it was the parents who were ultimately responsible for assuring that their child was appropriately dressed each day. Participants discussed putting up signs or sending home written reminders to dress children appropriately, a tactic that would require minimal interface with the parents about potentially contentious issues. Yet if teachers did not explain why appropriate clothes were necessary, these requests were often met with equally minimal success.

¶47 "Um, during the cold, we would get outside twice a week. You send notes home. 'Please dress your child properly, they need blah, blah, blah,' but it doesn't happen."

¶48 "You send notes home [that say] 'Please put gym shoes on,' [but] it does no good."

One participant described her success with a more passive-aggressive approach:

¶49 "I have an issue of going out without boots. It's snowy, they are gonna get wet. One day I send them home with wet, cold gym shoes. I got boots the next day!"

Others recounted successes in conveying their messages through the children, and in encouraging parents to talk directly with their children about going outside and the clothing necessary.

¶50 "When I have group I say, 'Would you share this with your parents when you get home because we are definitely going [outside]?' Then you have them excited and they'll come in all ready. 'See I got my gloves and everything'."

¶51 *The following quote was what a participant said to a mother who had requested that the teacher not take her child outside*: "'Think about your child. How's it gonna make your child feel, all of her friends are outside and she's inside?' [The parent had responded] 'I don't think she wants to go out because I don't take her outside.' [The teacher replied] 'Listen, let's talk to your child'."

Because participants felt that often times the inappropriate clothing items stemmed from a lack of parent awareness and understanding about the importance of outdoor play, several participants discussed the importance of parent education as an effective solution to clothing barriers.

¶52 "The disadvantage is when parents don't understand that kids get dirty. The parents are upset because they come home and their new clothes are dirty or they have leaves in their hair. But I consider that as an opportunity for parent education. I am not gonna quit doing it. We are not gonna quit using paint. I send home flyers and talk about what they're learning. I use it as an opportunity for parent education."

¶53 "You have to come back to the parent. This week our theme is community so we are going to be walking. This week our theme was snow. That was our science activity. You have to be able to give them the language to let them know you do understand why you did this. A lot of them appreciate that too."

In addition to educating parents about the many learning benefits of outdoor and active play, participants also found that if they could connect with the child's parents around a shared concern for the child's safety, they were more likely to be successful in encouraging parents to dress children appropriately.

¶54 "I have quite often asked parents to, not just with flip flops, maybe shoes with really slippery shoes. 'Your child is going to be climbing outside and they need some shoes that they can run and play safely.' Most parents just don't think about it."

¶55 "I once had a little girl in my class that she dressed so cute, adorable everyday, everything matching. She had these cute little flip flops and I hesitated to say something to her mom but I needed to for her safety. I said, 'I am really concerned about your child's safety. I don't want her to fall or get injured because she does a lot of climbing.' And so, she started sending her in gym shoes. So I think that was a positive thing for me to say that I was concerned."

¶56 "Some of my parents let their little girls wear hoops like this. I take upon myself I ask them, 'We do a lot of playing outside. Can you put on tiny studs?' And I haven't had any problem with parents doing that without having the policies."

Most teachers found parents receptive to their suggestions if teachers were able to explain why it was important for their child to be dressed to play and learn. In essence, teachers found that the most successful strategy for overcoming clothing barriers was to develop a positive rapport with parents. Most felt that this rapport and parental trust were essential for dealing with clothing issues.

¶57 "Acknowledge their feelings... 'I understand it is really upsetting. It's a new outfit, and it's upsetting. But If you remember, we talked about-- you might want to send old clothes'. Give out handouts about the benefit of active play, how much they're learning, the brain development and social development and every other kind of development, and that it's worth doing, even though it is a little bit of a hassle."

¶58 "So after a while, once you get a rapport with the parent, that does work and they usually will come around. Usually!!"

They achieved this rapport through handouts, highlighting areas of shared concern for the child's learning and safety, and multiple daily encounters over time. Participants also acknowledged that many parents became more relaxed and less over-protective as the child got older.

¶59 "I find that the younger--we have from 18 months to 36 months in my room--I find that when they come in at 18 months, they are very, very protective. If you go outside--- [parents will say] 'Well, it's only 25 degrees.' [*in response*] 'Well, yes, we go outside. We only stay out for 15 minutes but they do need to be outside.' They [parents] are not so receptive to that but as they get older, I don't know if it's just that the child is getting older or because they are not winning this battle. They're going outside so we might as well send them with everything they need. I don't know if we just wear them down or if they let go a little bit."

¶60 "I think when they're younger, especially the parents of a first child, they are learning too and they don't know that it's OK. Just that trust thing. A trusting relationship with the parent. When they first come to your center, it's like, [parent asks] 'You are taking them outside when it's only 25°? What are you talking about?' Once they trust you, and by the time they trust you their kid is older and preschool age."

## Discussion

Two major themes were expressed by virtually all participants: 1) children's clothing could serve as a barrier to their physical activity and 2) this clothing elicited conflict or tension between child-care providers and the children's parents, whom the providers felt were ultimately responsible for these clothing choices. These themes were unexpected, as they had not been widely reported particularly in the physical-activity literature.

Two recent qualitative studies[33,34], both from Australia, each briefly mentioned that the way children are dressed may impact their physical activity, although both provided minimal details as clothing was listed as one of many themes. O'Connor et al [33]was a study of family child-care home providers (not centers), and was published in the early education literature. Dwyer et al [34]was a study of child-care centers, but the clothing theme was classified as a "gender" issue, because participants mentioned it only as an issue for some Chinese and Middle Eastern parents of girls. Our findings would suggest that these practices are more widespread--both across different genders and cultural groups--as well as outside Australia. Furthermore, given that virtually all participants brought up clothing issues in response to general questions about barriers to children's physical activity, our findings would suggest that clothing issues may be a more important barrier than previously considered by policy makers and health researchers. Notably, because clothing issues have not been widely reported, they have not been considered either in recent studies[16-18,22] of environmental influences on children's physical activity in child care centers or in recent reviews [35,36] of the correlates of preschool-aged children's physical activity.

The purpose of our study was to explore reasons why physical activity levels may vary across different centers, and to use a methodology that applied no pre-conceived constructs, so that we could learn from those who were on the "front line" of children's activities in child-care settings. While it cannot be known to what extent these clothing items such as flip flops, dress clothes, and no coat truly impede children's physical activity, our findings suggest this questions warrants further study.

We were surprised by the pervasiveness of inappropriate clothing issues, as almost each participant provided anecdotes of one or a few children who consistently dressed inappropriately for play. We were especially surprised by stories of parents intentionally taking the child's coat to keep their child from going outside. While these may seem to be isolated issues with minimal impact, the importance of these seemingly minor factors may be profound. Inappropriate clothing may serve to limit physical activity even when children are given the opportunity to be outside. Similarly, only one or two children who are not dressed appropriately for cold weather may restrict daily the physical activity opportunities of the entire class if the teacher is unable to find warm clothing to loan, or another teacher to watch the child(ren) who are improperly dressed. This is particularly concerning because the default activities in child care centers appear to be primarily sedentary [11,12], as they present no safety hazards to children regardless of their dress. Differences in clothing practices may partly explain recent conflicting findings [16-18,22] about whether or not providing increased outdoor time can increase children's physical activity in child care. Specifically, if children are dressed in a way that impairs their activity, increasing their amount of exposure to the outdoors will not be able to overcome this impediment.

The deep level of tension between parents and child-care providers that these clothing discussions elicited was also somewhat surprising. When talking about parents, child-care providers ranged from empathetic, to skeptical/dubious, to almost hostile. Child-care providers displayed the most empathy when discussing how some parents of low socio-economic status may not be able to afford to leave a change of clothes in the center or proper winter clothing. Of note, the current wage of many assistant teachers (U.S.$8.39/hr) [32] would place them in the bottom quintile for U.S. household income. Several participants could relate to the difficulties of helping children get dressed for school in the morning, as 76% reported having children of their own and over half had used child care for their own children. Yet some expressed questions about why a parent would allow their child to wear jewelry or nice clothes to school, where getting dirty is an essential part of play and learning. Still others expressed almost veiled hostility for being asked to change a child's shoes in preparation to go outdoors, and being held responsible for lost jewelry. Conflict between parents and teachers has been reported previously, in that child-care providers felt parents would be the greatest barrier to implement new health promotion activities [37]. Our findings about the salience and ubiquity of these conflicts around clothing, and the limited success with which child-care providers have been able to change parents behaviors regarding children's attire, supports this assertion.

Our findings have a number of implications for both child-care providers and policy makers wishing to increase children's physical activity levels in child care. First, because participants expressed greater comfort with externally-imposed standards and policies to which they could refer parents and thus avoid a direct or personal confrontation, clear and specific policies about children's clothing should be developed and implemented. Ideally these policies would be grounded in state licensing or national best practice manuals, such as Caring for our Children Health and Safety Standards for Out-of-Home Child Care [38], as the center directors also appreciated having externally-imposed guidelines to back up their center practices. Centers should require that children come to school dressed appropriately for active play. Clothing should permit easy movement (not too loose and not too tight) and footwear should provide adequate support for running and climbing. Appropriate clothing would include:

1. gym shoes or sturdy gym-shoe-equivalent (no flip flops or other types of shoes that can come off while running, or that provide insufficient support for climbing);

2. no "dress clothes" or special outfits or that are not allowed to get dirty; and

3. appropriate clothes for the weather, including heavy coat, hat, and mittens in the winter, raincoat and/or boots for the rain, and layered clothes for climates in which the temperature can vary dramatically on a daily basis.

Directors should consider outlining the importance and benefits of outdoor play for children's development in the parent handbook, and referring to these benefits frequently in discussions with parents. A policy prohibiting large and/or expensive jewelry would also seem reasonable, both for safety and liability reasons.

The issues related to adequate coats, hats, and gloves in cold temperatures may be more difficult to overcome, as parents may inevitably continue to forget, and some low-income parents may not be able to afford proper clothing for all temperatures. Center directors should be aware of how the "car culture" may contribute to parents' forgetfulness and be sensitive to these issues when addressing this with parents. Directors should also consider keeping extra hats, gloves, and coats available to loan on cold days. On rainy days, directors should consider using or constructing covered areas to the playground, or providing raincoats and boots. Centers with gross-motor-rooms or muscle rooms could use these rooms on days when weather or improper dress does not permit play outdoors. Directors may also find that parents may respond to more specific guidelines, e.g., "Children will be taken outside every day for at least 15 minutes when the temperature exceeds 21°F; please provide hat and gloves for all temperatures below 50°F; and a coat or jacket for all temperatures below 60°F." Such specific guidelines would take the guesswork out of dressing the child in the morning, and would make it more likely that all children are dressed to go outside. As for children who come overdressed, the fact that some centers require that all children wear all layers of clothing was a surprise. This practice may not be common in other areas where the temperature can vary 30-40°F in a single day. Nevertheless, centers that have these policies may need to consider revising them and allowing the teacher and child to use their best judgment.

### Limitations

Although we tried to recruit participants with a range of experiences and perspectives, participation was voluntary and there may have been a selection bias; people who elected to participate all seemed relatively interested in children's physical activity opportunities. The prevalence of reported perceptions about clothing barriers and the frequency of these clothing practices cannot be determined from a qualitative study. However, the content of international blog posts on the New York Times website reporting the study findings [31] suggested these clothing practices are also seen in other parts of the country and world. A strength of undertaking this study in Cincinnati is that the city is located in a temperate zone with distinct seasons that require different types of clothes. Previous studies in more moderate climates such as in coastal California [22] may not have been able to determine the potential effect of inappropriate clothing. Nearly all of the participants were female, but this is reflective of the child-care work force [32]. Participants were either Caucasian/white or African-American/black, with no other ethnic/racial groups represented, consistent with the predominant ethnic/racial groups in Hamilton County. Future studies are needed to better understand barriers to children's physical activity that may be particular to certain U.S. and non-U.S. cultural groups that were not well represented in this study including Latinos, Asians, and American Indians.

## Conclusions

This is the first study to thoroughly examine and identify as a major theme children wearing inappropriate clothing as potential barrier to their physical activity in child care. Child-care providers in these focus groups reported that children commonly wear inappropriate clothing such as flip flops, no coat/hat/gloves in winter, and dress clothes with instructions not to get them dirty. We found that these clothing issues were a significant source of tension between parents and child-care providers. With three-quarters of children in some form of child care, and increasing evidence that young children spend very little (2-3%) of their time in these settings in moderate or vigorous physical activity [12], it is imperative to understand what barriers exist in these settings to limit children's physical activity. Our findings suggest that inappropriate clothing is a salient--and possibly considerable--barrier to children's physical activity. More research is needed to determine the prevalence of these clothing practices, and the extent to which they may influence children's physical activity. Specifically, researchers may want to examine if a child wearing flip flops is less active than when wearing gym shoes, or alternatively if centers that permit the wearing of flip flops or dress clothes have significantly lower physical activity levels than those that do not, other things being equal. Our findings suggest that center directors and policy makers should consider devising clear and specific policies for the types of clothing that will be permitted in these settings so that children's active play opportunities are not curtailed. To ensure compliance with these policies, some parents may need further education about the importance and benefits of outdoor play for children's development. Centers may also consider employing pragmatic solutions to clothing issues, such as keeping extra coats or hats to loan, or employing the use of indoor spaces that can be customized to permit vigorous play.

## Competing interests

The authors declare that they have no competing interests.

## Authors' contributions

KAC conducted and supervised the study, attended all focus groups, conducted half of the interviews, assisted with thematic analysis, and drafted the paper. SNS facilitated all focus groups, conducted half of the interviews, assisted with thematic analysis, and contributed to the drafting of the paper. CAK assisted with thematic analysis, editing the transcripts, and edited the paper. BES was involved in the design of the study and contributed to drafting the paper. HKK was involved in the design of the study, provided guidance in conducting the study and in data analysis, and contributed to the drafting of the paper. All authors read and approved the final manuscript.

## Authors' information

KAC is an academic general pediatrician with experience conducting health-related research in child care settings. SNS is a consultant with expertise in qualitative and survey methods, and experience conducting obesity-related research. CAK is a research assistant with a Bachelor's degree in psychology and experience working in a child care center. BES is a research psychologist with experience in obesity-related research and the built environment. HJK is a nutritional epidemiologist with research experience in children's physical activity, dietary intake and bone health.

## Appendix - Sample questions used in focus groups that elicited comments about clothing*

**1**. How are **outside games **different than **inside games**?

◦ Which do you enjoy more? Why?

◦ How are **outside rules **different from **inside rules**?

**2**. What are some possible **benefits **to children being outside?

**3**. What are some possible **disadvantages **to children being outside?

**4**. What are some things you like/dislike about your **playground**? What about the children, what do they like/dislike about the playground?

**5**. What **types of things keep you from using your playground **sometimes? *Probe on the following in whatever order the participants mention them*

◦ What types of **weather **keep children from going outside or using your playground?

◦ What about the **parents**?

▪ *Additional probes if needed:*

• Have parents ever **discouraged **you from going outside or using the playground?

◦ How do you handle that?

◦ How do you feel about that?

• Do parents ever **encourage **you to take their children outside?

**6**. What kind of **policies **does your center have about using the playground, including **weather conditions**, playground **schedule?**

◦ *In later focus groups the following additional questions were asked, only after the participants brought up clothing issues:*

▪ What sort of policies does your center have about children wearing **flip flops**? **Jewelry**? **Any other clothing **that restricts activity?

▪ What does your center do when children are not properly dressed for the weather?

* For each of the questions, non-specific and non-leading probes were used to follow up on any ideas expressed. Examples of these probes were "Tell me more about that," or "Can you provide an example?"
